# A microsatellite-based consensus linkage map for species of *Eucalyptus *and a novel set of 230 microsatellite markers for the genus

**DOI:** 10.1186/1471-2229-6-20

**Published:** 2006-09-22

**Authors:** Rosana PV Brondani, Emlyn R Williams, Claudio Brondani, Dario Grattapaglia

**Affiliations:** 1 EMBRAPA Recursos Genéticos e Biotecnologia, CP 02372, 70770-970 DF Brasilia, Brazil; 2 Department of Cell Biology, Universidade de Brasília UnB, DF, Brasília; 3 EMBRAPA Arroz e Feijão, CP 179, Goiânia GO 74001-970, Brazil; 4 CSIRO Forestry and Forest Products, POBox E4008, Kingston ACT 2604, Australia; 5 Graduate Program in Genomic Sciences and Biotechnology, Universidade Catolica de Brasília, 70790-160 DF Brasilia, Brazil

## Abstract

**Background:**

Eucalypts are the most widely planted hardwood trees in the world occupying globally more than 18 million hectares as an important source of carbon neutral renewable energy and raw material for pulp, paper and solid wood. Quantitative Trait Loci (QTLs) in *Eucalyptus *have been localized on pedigree-specific RAPD or AFLP maps seriously limiting the value of such QTL mapping efforts for molecular breeding. The availability of a genus-wide genetic map with transferable microsatellite markers has become a must for the effective advancement of genomic undertakings. This report describes the development of a novel set of 230 EMBRA microsatellites, the construction of the first comprehensive microsatellite-based consensus linkage map for *Eucalyptus *and the consolidation of existing linkage information for other microsatellites and candidate genes mapped in other species of the genus.

**Results:**

The consensus map covers ~90% of the recombining genome of *Eucalyptus*, involves 234 mapped EMBRA loci on 11 linkage groups, an observed length of 1,568 cM and a mean distance between markers of 8.4 cM. A compilation of all microsatellite linkage information published in *Eucalyptus *allowed us to establish the homology among linkage groups between this consensus map and other maps published for *E. globulus*. Comparative mapping analyses also resulted in the linkage group assignment of other 41 microsatellites derived from other *Eucalyptus *species as well as candidate genes and QTLs for wood and flowering traits published in the literature. This report significantly increases the availability of microsatellite markers and mapping information for species of *Eucalyptus *and corroborates the high conservation of microsatellite flanking sequences and locus ordering between species of the genus.

**Conclusion:**

This work represents an important step forward for *Eucalyptus *comparative genomics, opening stimulating perspectives for evolutionary studies and molecular breeding applications. The generalized use of an increasingly larger set of interspecific transferable markers and consensus mapping information, will allow faster and more detailed investigations of QTL synteny among species, validation of expression-QTL across variable genetic backgrounds and positioning of a growing number of candidate genes co-localized with QTLs, to be tested in association mapping experiments.

## Background

Eucalypts are the most widely planted hardwood trees in the world occupying globally more than 18 million hectares [1]. While *E. globulus *is the premier species for temperate zones plantations in Portugal, Spain, Chile and Australia, elite hybrid clones involving *E. grandis *and *E. urophylla *are extensively used by the pulp and paper industry in tropical regions of Brazil, South Africa, India and Congo because of its wood quality, rapid growth, canker disease resistance and high volumetric yield [2].

Genetic mapping became accessible to several forest tree species in the beginning of the 90's based on the combination of the speedy and inexpensive generation of dominant RAPD and AFLP markers and the pseudo-testcross strategy in two-generation pedigrees [3,4] or the use of the haploid genetics of conifers [5-7]. Concomitant to this development, linkage maps of co-dominant markers led to the construction of integrated RFLP maps for a few species [8,9] and the possibility of comparative mapping [10,11]. However it soon became clear that true advancements in QTL validation across pedigrees and eventually marker assisted selection in forest trees, would strongly depend on the availability of higher throughput, higher polymorphism typing systems such as microsatellites, organized in dense genetic maps [12,13]. In the last few years a number of studies reported genetic maps for forest trees built with combinations of several hundred RAPD and AFLP markers together with some tens of EST, genes and microsatellites (e.g. [14-20]). Linkage maps with around one hundred microsatellites were reported for *Pinus taeda *[21] and *Populus *[22]. However to allow a more precise comparison of QTL position and validation of putative QTL across pedigrees larger sets of microsatellites are clearly necessary.

One hundred thirty seven autosomal microsatellite markers have been published to date for species of *Eucalyptus*, including twelve from *E. globulus *named with the prefix EMCRC [23], eight from *E. nitens*, eight from *E. sieberi*, 26 from *E. globulus *and 13 from *E. leucoxyon *named respectively with prefixes En, Es, Eg and El [24-27] and 70 from *E. grandis *and *E. urophylla *named EMBRA [12,15]. Recently a set of 35 chloroplast DNA microsatellites were developed based on the full cp-DNA sequence of *E. globulus *[28]. Microsatellite transferability across species of the subgenus *Symphyomyrtus *varies between 78 and 100% depending on the section to which they belong. It still remains around 50 to 60% for species of different subgenera such as *Idiogenes *and *Monocalyptus *and goes down to 25% for the related genus *Corymbia *[29]. Microsatellite comparative mapping data has also shown that genome homology across species of the same subgenus *Symphyomyrtus *is very high not only in terms of microsatellite flanking sequence conservation, but also marker order along linkage maps [30]. Although 70 microsatellites were mapped on a framework map of RAPD markers [15], 34 on a framework of AFLP markers [30], and 40 on a combined RFLP and candidate gene map [17], the genus *Eucalyptus *still lacks a more comprehensive genetic map built exclusively with microsatellite markers.

Several QTL mapping reports have demonstrated the existence of major effect QTLs for a number of silviculturally and industrially relevant traits in *Eucalyptus *[31-39]. Recently, QTL analysis of transcript levels of lignin-related genes showed that their mRNA abundance is regulated by two genetic loci co-localized with QTLs for growth, suggesting that the same genomic regions are regulating growth, lignin content and composition [40]. In a subsequent study it was also shown that a single eQTL identified explained up to 70% of the transcript level variation for over 800 genes and that hotspots with co-localized expression QTLs were identified typically containing genes associated with specific metabolic and regulatory pathways, suggesting coordinated genetic regulation [41].

Although the number of QTL detection reports in *Eucalyptus *has grown and become increasingly sophisticated, the large majority of the mapped QTLs have been localized on RAPD or AFLP maps so that it is essentially impossible to compare positions of QTLs for the same or correlated traits, seriously limiting the long term value of such QTL mapping efforts for genomics and breeding applications. Exceptions are QTL studies where transferable markers such as a few microsatellites [30,38] or candidate genes [14,38] were also mapped so that it is at least possible to make a coarse preliminary comparison of QTL locations at the linkage group level. Especially in the genus *Eucalyptus *where breeders worldwide take advantage of the interspecific genetic variation for wood properties and disease resistance through hybridization, the availability of a robust, genus-wide genetic map with highly transferable microsatellite markers has become a must for the effective advancement of genomic undertakings including QTL validation across pedigrees, co-localization of QTL and candidate genes for guiding association mapping experiments, positional cloning of QTLs and eventually marker assisted selection.

This work reports on the construction of a consensus genetic linkage map covering all 11 linkage groups of *Eucalyptus *including a total 234 mapped loci making it, to our knowledge, the most complete genetic map of *Eucalyptus *and of a forest tree to date based exclusively on interspecific transferable microsatellites. Besides the linkage map, a comprehensive set of 230 novel microsatellite markers are reported and a subset of 35, selected as anchor loci, were characterized for mapping information content. Finally, based on a set of shared microsatellites with other *Eucalyptus *mapping studies, a further set of 41 microsatellites, candidate genes and QTLs for wood and flowering traits are assigned to the consensus map making it the first consolidated source of existing linkage information for species of *Eucalyptus*.

## Results

### Microsatellite polymorphism in *Eucalyptus*

**Figure 1:**
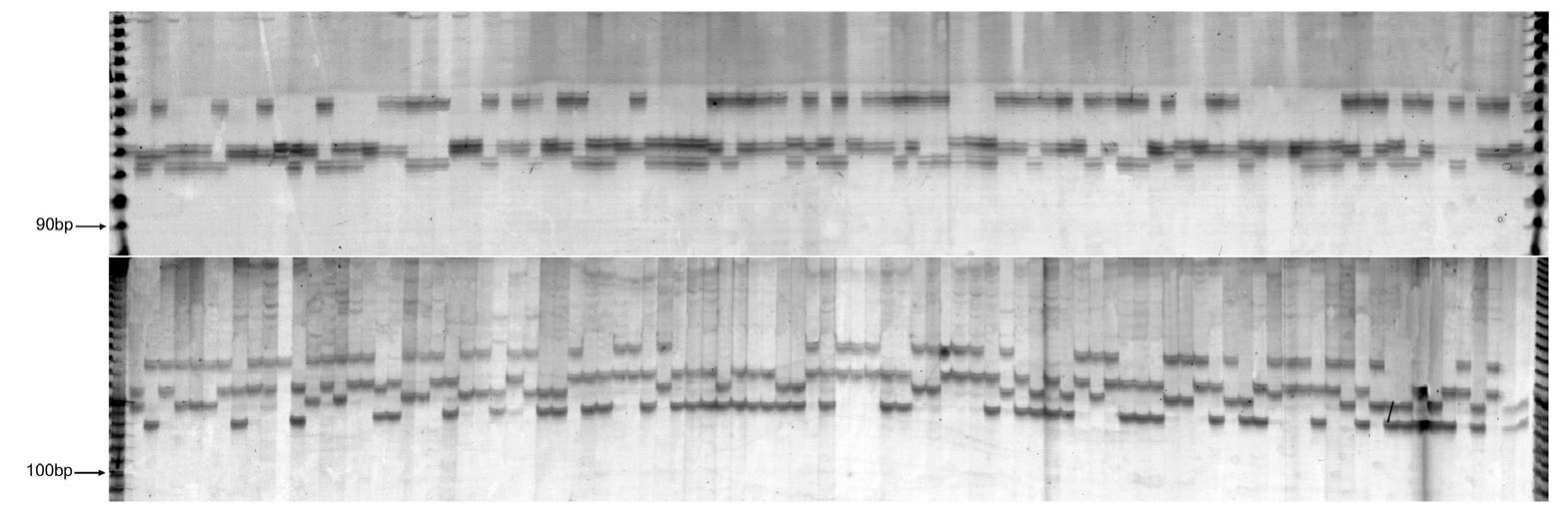
Inheritance and segregation of fully informative microsatellites in *Eucalyptus*. Denaturing polyacrylamide gel resolution and detection by silver staining of markers EMBRA180, (top panel), EMBRA192 (bottom panel). First and last lanes contain the 10 bp ladder size standard, Invitrogen) with sizes of two fragments indicated in base pairs; lane 2 and 3 are the two parents, *E. grandis *G44 and *E. urophylla *U28, followed by 92 F1 progeny individuals.

**Figure 2:**
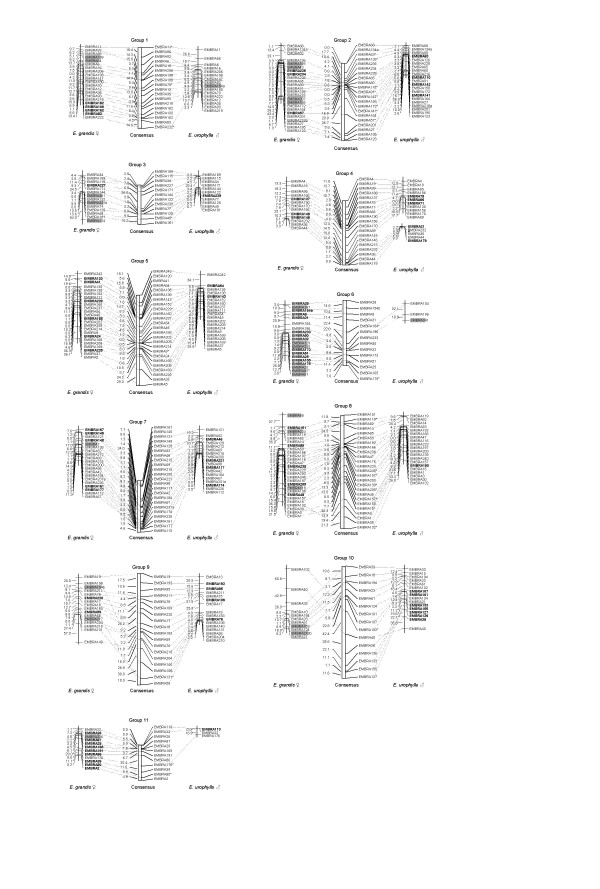
Parental and consensus maps of *Eucalyptus *involving a total of 234 microsatellite markers on 11 linkage groups. Markers in bold were mapped on one of the parental maps and on the consensus map; markers in grey boxes were mapped on one of the parental maps but not on the consensus map; underlined markers were mapped on both parental maps but not on the consensus map. Asterisks indicate markers with segregation distortion. Dotted lines connect the same markers in different maps, so that crossing lines indicate changes in locus ordering among maps. Distances in centiMorgan, (cM) Kosambi are indicated on the left of each linkage group.

From ten *E. grandis *enriched libraries for poly-(AG) and poly-(AC) repeats, a total of 450 primer pairs complementary to microsatellite flanking sequences were designed. Seventy of them were previously mapped, characterized and had their primer pairs sequences published [12,15]. In this work, the remaining three hundred-eighty primer pairs were tested for robustness of PCR amplification followed by screening for polymorphism, inheritance and segregation in the mapping population (Figure 1). Although all primer pairs were designed to amplify at the same annealing temperature (56°C), some optimization was necessary between 48°C and 60°C, to improve genotype interpretation. From the 380 markers evaluated, 230 generated robust and easily interpretable genotypes that could satisfactorily be used for individual genotyping and genetic mapping. The remaining 150 primer pairs either did not amplify, even reducing the annealing temperature, or amplified complex patterns of segregation possibly due to non-specific amplification. Out of the novel set of 230 markers, 167, i.e. 73% were heterozygous in one or both parents allowing the analysis of segregation and 63 (27%) did not segregate in this particular interspecific pedigree. The 167 polymorphic microsatellite marker primer pairs amplified 171 segregating loci as four primer pairs (EMBRA134, EMBRA154, EMBRA218, EMBRA231) amplified duplicated loci indicated with letters (a) and (b) on the map (Figure 2). Locus duplication was inferred by the fact that the two loci amplified with the same primer pair mapped to different linkage groups. Additional file 1 summarizes all the information for the 230 markers reported in this study (EMBRA71 through EMBRA395) plus those published earlier (EMBRA1 through EMBRA70) [12,15] including the Genbank accession number of the original sequences from which the microsatellite primer pairs were designed.

### Microsatellite segregation and linkage analysis

The 237 segregating markers used in the construction of the consensus map (167 from this study plus 70 published earlier [12,15] totaled 241 segregating loci due to the four locus duplications detected that could be mapped. Out of these 241 loci, 234 could be mapped with high confidence (Figure 2). A descriptive summary of the main attributes of both parental and consensus maps, organized by linkage group, was compiled (Table 1). Out of the 234 markers mapped in this study, 74 were only female (*E. grandis*) informative and 32 only male (*E. urophylla*) informative. A total of 128 (55%) were fully informative, i.e. segregated from both parents with a total of three or four different alleles. No markers were observed segregating 1:2:1, i.e. equally heterozygous in both parents. However only 122 out of the 128 fully informative markers could be placed on the consensus map. Six markers, although segregating from both parents, could not be positioned on the consensus map. These markers, indicated in the map with an underline (Figure 2), were EMBRA21, EMBRA147, EMBRA055, EMBRA111, EMBRA216 and EMBRA218a. For these six markers at least one null allele was detected in one of the parents, what might have contributed to impede their integrated mapping. The presence of null alleles, i.e. the non-amplification of one or both alleles at the locus, was in fact observed in 20 out of the 241 segregating marker loci. Twelve out of the 20 microsatellites with null alleles were homozygous null in *E. urophylla *but amplified both alleles in *E. grandis*; four markers had at least one null allele in both parents with *E. urophylla *being homozygous null at two loci; three were heterozygous null in *E. urophylla *and only one marker had a null allele in *E. grandis *and not in *E. urophylla *(Table 2). The overall frequency of null alleles was therefore 5 in 241 loci for *E. grandis *and 19 in 241 for *E. urophylla*. A higher observed heterozygosity was observed in the *E. grandis *parent tree compared to *E. urophylla*. In total, 208 markers were heterozygous in *E. grandis *and 166 in *E. urophylla*. Even discounting the 14 microsatellite markers that did not amplify in *E. urophylla *(were homozygous null), *E. grandis *would still display 194 heterozygous markers, i.e. 17% more than *E. urophylla*. This difference in observed heterozygosity is most likely due to the variable inbreeding status of the particular parents and not a species characteristic.

### Linkage map construction

At the statistical stringency adopted for linkage analysis, the maternal *E. grandis *map had a total of 202 markers organized into 11 linkage groups and the paternal *E. urophylla *map had a smaller number of markers, 160, in 12 linkage groups, one more than the expected number (n = 11). This extra group is a set of three markers that mapped at the end of linkage group 4 in *E. urophylla *at a LOD threshold lower than 3.0. These three markers most likely belong to this group but will require more markers to bridge them consistently to group 4. Seven markers although informative, remained unlinked on the map built only with microsatellites. These markers were: EMBRA94, EMBRA96, EMBRA103, EMBRA163, EMBRA178, EMBRA190, from the novel set of 230 markers reported in this study, and EMBRA62 previously mapped to the RAPD marker framework of linkage group 11 of *E. urophylla *in Brondani *et al*. [15]. EMBRA62, remained unlinked to the map constructed in this study, possibly due to the very low density of microsatellite markers mapped on group 11 for *E. urophylla *(Figure 2).

The female map with 202 markers covered an observed length of 1,814.5 cM with a mean distance between adjacent markers of 10.7 cM calculated as the arithmetic mean of the map distances between adjacent markers in each linkage group and not just simply by dividing the total map length by the number of markers. For the *E. urophylla *male map the total recombination map distance covered was 1,133.4 with an average distance between adjacent markers of 9.2. A paired t-test revealed no significant difference in the mean recombination fraction between adjacent markers markers (10.7 for *E. grandis *and 9.2 for *E. urophylla *– Table 1) when comparing the two parental maps. The larger observed total map length of *E. grandis *is therefore most likely due to the larger number of markers (202) mapped when compared to *E. urophylla *(160). The consensus map had an observed length of 1,567.7 cM and a mean inter-marker distance of 8.4 cM. A total of 19 map intervals with a genetic distance greater than 20 cM were observed, scattered throughout almost all linkage groups, except groups 1 and 4, but only 5 out of the 19 intervals were larger than 30 cM, indicating a relatively homogeneous map coverage obtained when using exclusively microsatellites markers. Clustering of markers was observed on both parental maps and consequently on the consensus map, particularly on groups 2, 5 and 7, although no formal test for clustering was carried out. Interestingly, however, clustering of markers had also been observed on these same linkage groups when built with RAPD markers using this same set of progeny [4], suggesting a biological basis for this occurrence.

The estimated genome length (G_est_) of the consensus map was 1,683 cM while the observed length (G_obs_) was 1,567.7 (Table 1), resulting in an observed genome coverage C_obs _= 93%. The theoretical expected genome coverage for the consensus map obtained using the equation by Lange and Boehnke (1982) was estimated as C_exp _= 88.6%.

The number of common markers between the two parental maps was heterogeneous along the eleven linkage groups. For example while for linkage group 1 with 22 markers mapped, 14 were mapped in both parents and on the consensus map, for linkage group 6 with 21 markers only 2 were mapped on both parental maps and on the consensus (Figure 2).

A Chi-square revealed that out of the 241 microsatellite loci, 29 (12%) deviated from the expected 1:1:1:1 or 1:1 segregation ratio at alpha ≤ 0.05 but only one (EMBRA81) remained significant after applying a Bonferroni correction. All these markers but EMBRA81 on group 6 could be confidently placed onto the consensus map. Sixteen distorted markers clustered mainly into two linkage groups (group 2 and 8) and the remaining 14 were scattered across eight linkage groups (Figure 2).

### Comparative mapping

The 122 fully informative markers mapped, allowed a robust identification of homologous pairs of linkage groups of the *E. grandis *and *E. urophylla *genomes. Without exceptions, all microsatellite markers that mapped on the same linkage group in one species also did so in the other species. We observed that despite the fact that approximately 17% of the two-point estimates of recombination frequency observed for the same pairs of microsatellite markers differed considerably (on average by 25%), conservation of locus order was observed between the two parental maps, with 82% of the markers mapping with the same linear order along the individual linkage groups. This conserved linear ordering can be easily visualized by the dotted lines connecting the parental maps with the consensus map (Figure 2). Occurrences of ordering change were rare, involving one or two markers mapping in a different order in relation to the rest on the same group, such as EMBRA92 on group 1, EMBRA30 and EMBRA132 on group 8 or EMBRA156 and EMBRA170 on group 4. A greater level of marker order scrambling was observed on groups 9 and 10, possibly due to the fact that several markers on this group segregated from one or the other parent only. In the *E. grandis *map, from 172 markers available for comparisons, 153 (89%) kept the same order in the consensus map. In *E. urophylla*, out of the 152 markers, 140 (92%) kept the same order as in the *Eucalyptus *consensus map. Out of the 122 markers mapped on all three maps, thus comparable, 85% of them kept the same order. No evidence of rearrangement of chromosomal block between the two species was found. As expected, the size of the consensus map had an intermediate size (1,567 cM) between the female (1,814 cM) and male (1,133 cM) parental maps.

### Microsatellite polymorphism

Genetic diversity for 35 markers selected as anchor loci due to their transferability and ease of interpretation was determined to guide future mapping experiments (Additional file 2). A similar average number of alleles at these microsatellite markers was found in the two species, 10.61 ± 3.05 for *E. urophylla *and 10.66 ± 2.59 for *E. grandis*, with 50% of the alleles shared between them and the other 50% appearing exclusively in one or the other species most likely due to sampling effect although suggesting that important differences in microsatellite allele frequencies do exist between these two species. These frequency differences increase the probability of detecting markers segregating in a fully informative fashion, thus more informative for QTL mapping. The highest diversity was observed for marker EMBRA201 – originally a 17 dinucleotide repeat long – displaying 22 alleles in 64 chromosomes sampled. The total number of alleles detected in the two species combined ranged from 8 to 22, with the maximum allele size of 320 bp and minimum of 75 bp. The average observed heterozygosity of the 35 loci was around 66%, while the expected heterozigosity was higher, around 85%. These results are in agreement with a small level of selfing and/or related matings in natural populations of *Eucalyptus*.

### Compilation of microsatellite linkage information

Based on the EMBRA markers mapped in other linkage studies in *Eucalyptus*, it was possible to establish the homology among the linkage groups of this consensus map and those of other linkage maps published (Table 3). This analysis allowed the linkage group assignment of other 41 microsatellites developed in other laboratories and from different *Eucalyptus *species: four EMCRC from *E. globulus*, 26 microsatellites Eg from *E. globulus *(Eg), seven from *E. nitens *(En) and four from *E. sieberi *(Es). Furthermore 18 candidate genes for wood fiber and floral traits, previously mapped by Thamarus et al. [17] could also be assigned to the groups of this consensus map.

## Discussion

Prior to this work, important advances were made in the construction of genetic maps of species of *Eucalyptus*. Although some RFLP markers were mapped in *E. nitens *[9] and *E. globulus *[17], the most extensive genetic mapping data has been accumulated mainly with dominant RAPD and AFLP markers [4,42-45]. While RFLP markers are useful for comparative mapping purposes across individuals, species and even more distant taxa, high throughput genotyping, probe distribution and maintenance are difficult. On the other hand RAPD and AFLP markers while allowing the generation of several hundred markers and providing very good genome coverage, have limited information content and are almost useless for comparative mapping studies and QTL validation across pedigrees and species. The novel set of 230 microsatellite markers reported herein, summed to the 70 markers reported earlier [15], totals a relatively large set of 300 microsatellites that should allow significant advances in *Eucalyptus *genetic research. Furthermore, the linkage map presented, involving 234 mapped loci, spans an estimated ~90% of the recombining genome of *Eucalyptus*, making it the most comprehensive genetic linkage map of a forest tree to date based exclusively on microsatellite markers.

### Microsatellite development

Consolidating all the development and screening data since our initial studies [12], from 450 primer pairs designed, we obtained 300 operationally usable markers, i.e. a final efficiency of marker development of 67%. Out of these 300 microsatellites, we were able to detect polymorphism and Mendelian segregation at 237, i.e. 79%, in this particular mapping population. Although no published study is yet available making a detailed evaluation of large sets of microsatellite markers in other *Eucalyptus *pedigrees, a similar proportion of informative markers could be obtained when genotyping tropical eucalypt progenies within the section *Latoangulatae *of subgens Symphyomyrtus. In fact Missiaggia *et al*. [46] were able to easily select 100 informative microsatellites distributed throughout the *Eucalyptus *map in a cross of *E. grandis *× *E. urophylla *hybrid parents when mapping QTL for early flowering. Moving microsatellites to other commercially important species such as *E. globulus *(section *Maidenaria*) or *E. camaldulensis *(section *Exsertaria*) the issue becomes one of transferability first and then information content. While in an earlier study, based on 100 microsatellites, we indicated a transferability of 78% from *E. grandis *to *E. dunni *(section *Maidenaria*) [29], estimates of transferability and information content are still limited to reports based on mapping a few microsatellites in *E. globulus *[17,44], *E. camaldulensis *[47] and a slightly more extensive study involving *E. globulus *and *E. tereticornis *[30]. These studies taken together suggest, however, that transferability of these 300 microsatellites within *Symphyomyrtus *and across section, particularly *Maidenaria *where *E. globulus *and *E. nitens *belong, should remain around 80%. Once robustly transferred, it is likely that polymorphism should be detected at a rate similar to the one in this study, i.e. 70 to 80%. This entire set of 300 EMBRA microsatellite markers in addition to the 67 published by other groups should therefore allow positioning 200 or more informative markers on any segregating family involving any of the most planted species of *Eucalyptus*.

Besides microsatellites, other sources of genetic markers such as EST, gene-based and SNP have been [14,17] and will likely be increasingly mapped in *Eucalyptus *providing important anchor loci and candidate genes for positional cloning efforts as well as association mapping experiments. These markers, however, will demand high throughput typing techniques based on single nucleotide polymorphism assays to be able to be widely used across pedigrees. Other sources of microsatellites will also be important to sample regions of the genome that have not been contemplated so far. For example, 93 operational microsatellites were derived from a sample sequencing study of 3 megabases of shotgun DNA of *Eucalyptus grandis *[48]. EST derived microsatellites are another important source of novel microsatellites. Exploiting the large EST databases constructed in the Genolyptus project [49] we have been rapidly expanding the number of markers currently being mapped on a set of reference pedigrees. Three important aspects must be pointed out in this respect: (1) EST-derived microsatellites will efficiently complement the ones developed from enriched genomic libraries sampling different portions of the *Eucalyptus *genome; (2) microsatellites into transcribed regions, specifically in untranslated regions such as 5'-UTR, should be evolutionarily older than those in noncoding regions and thus are expected to be more polymorphic as reported in a survey of some major monocots and dicots species [50]; (3) genetic mapping of EST-derived microsatellites will enrich the map with transcriptional information opening up the perspective of co-localization of QTLs and candidate genes in regions of higher recombination.

### Eucalyptus microsatellite features

Fully informative markers that allow integration of the parental maps have been always considered key elements for a more detailed examination of interaction among alleles at QTL in forest trees [51]. The pseudo-testcross design and marker full informativeness are, evidently, mutually exclusive. RFLP based markers do reveal such 3 or 4-allele segregation, however not to an extent that allows map-wide analysis of such detailed QTL properties. In *Eucalyptus*, 33% and 36% of fully informative RFLP markers were detected respectively [9,17]. We originally reported 80% of fully informative microsatellite markers based on a mapped set of 20 markers [12], a proportion biased upward due to a stronger selection of polymorphic markers that was later revised to a more realistic 60 to 70% [15]. In this study 128 of the 234 mapped microsatellites (55%) were fully informative and no markers segregating in a 1:2:1 configuration were detected. Thamarus *et al*. [17] found a similar proportion of fully informative markers, 24 in 40 (60%) using a different set of microsatellites in an intraspecific *E. globulus *pedigree and also did not detect any marker segregating 1:2:1. These results taken together, and now based on a larger set of mapped markers from different sources, indicate that around 60% of a screened set of *Eucalyptus *microsatellites should segregate in a fully informative fashion. Furthermore the fact that the pedigree used in this study is interspecific, should not significantly increase the proportion of fully informative markers due to the fact that *E. grandis *and *E. urophylla *although separate species, belong to the same section (*Latoangulatae*).

Null alleles at microsatellites is a general occurrence reported in essentially all species where two-generation analysis required for genetic mapping or paternity determination have been carried out (reviewed in [52]). In this study, the overall occurrence of null alleles, was inferred in 20 (8%) out of the 241 segregating marker loci. Most markers were in fact homozygous null in *E. urophylla *but amplified both alleles in *E. grandis *(Table 2). The overall frequency of loci displaying null alleles was only 2% in *E. grandis *while 8% in *E. urophylla *most likely reflecting the fact that microsatellites were originally developed from an enriched *E. grandis *library. No other genetic mapping report of *Eucalyptus *mentions the frequency of microsatellite markers with null alleles. However the result of this study suggests that even for microsatellites deemed transferable across species the frequency of null alleles should increase as we move to species more distantly related to *E. grandis*. The presence of null alleles in heterozygosity is not a problem for the construction of the separate parental maps. By scoring the two segregating alleles in a binary fashion it is sufficient to observe only one allele while the other is scored as null. However, for the construction of the consensus map, the complete genotypic class information is necessary to perform the analysis, resulting in the exclusion of loci with one or more than one null allele. In fact all six fully informative markers with one or more null alleles segregating could not be positioned on the consensus map. It will be interesting and important to accumulate data on genetic mapping and null allele frequency at all the microsatellite available for *Eucalyptus*, so as to arrive to a robust set of markers with low frequency of sequence polymorphism in the microsatellite priming sites. EST derived microsatellites will likely supply a good source of such markers.

Combining the linkage information derived from the two parental maps and the consensus map, a total of 234 marker loci were consistently mapped at LOD 3.0. A larger number of markers segregated and were mapped on the *E. grandis *map (202) than on the *E. urophylla *map (160). In principle this should be due to a higher level heterozigosity in the *E. grandis *parent tree. However, previous survey of randomly distributed sequence polymorphism with RAPD markers in these same two parents did not show significant difference in the number of segregating markers with 272 heterozygous markers from *E. grandis *and 286 from *E. urophylla *assayed with the same set of arbitrary sequence primers [4] The observed difference in mappable microsatellites is most likely due to the incomplete transferability of markers between these two species as they were originally developed from a *E. grandis *library. Fourteen microsatellites did not amplify in *E. urophylla *(Table 2). In addition some *E. grandis *microsatellite loci, although yielding amplicons in *E. urophylla *at the same locus defined by the flanking primer sequences, could be bearing modified simple sequence repeats in *E. urophylla*. This occurrence, i.e. amplification of a PCR product but absence of sequence polymorphism has been observed when attempting to transfer microsatellites across related species [53,54]. In a microsatellite transferability study between *Quercus *and *Castanea*, despite the high sequence identity at the flanking regions observed for 14 loci mapped in corresponding linkage groups, the repeat motif in the non-source species was in some cases shortened and/or modified [55].

### Consensus map construction

The effect of merging parental maps in a consensus map has been a matter of debate about the final quality of locus ordering and estimates of recombination fraction. For example, while Maliepaard *et al*. [56] suggested that merging linkage maps with large differences in recombination rates can result in incorrect marker orders in the integrated map, Lespinasse *et al*. [57] found that even with significant differences in recombination between the parental meiosis, the merged maps displayed only slight differences in marker order. In order to better evaluate the effect of combing segregation data form both parents in a single map, we chose to present the separate parental species maps built using a widely used approach and software (pseudo-testcross and Mapmaker [4]) and compare them with the consensus map resulting form the integrated segregation data analyzed with Outmap. Although 17% of the two-point estimates of recombination frequency for the same pairs of microsatellite markers differed considerably between the two parental maps (on average by 25%), an overall analysis showed no significant difference in the mean recombination fraction between adjacent markers when comparing the two parental maps. This result indicates that the reported map distances between adjacent markers on the consensus map should be adequate average estimates. As expected, the total size of the consensus map was thus intermediate between the two parental maps. The consensus map had an observed length of 1,567.7 cM and a mean inter-marker distance of 8.4 cM. This mean distance does not fall between the mean inter-marker distances of the two parental maps (10.7 and 9.2) as one could expect (Table 1). The consensus map is, in fact, a newly constructed map based on the consolidation of segregating markers inherited from both parents. While the total map distance of the consensus was a close average between the two parental maps, the total number of markers mapped on the consensus map is larger or at least equal to the individual parental maps, thus resulting in a denser map and a reduced average inter-marker distance.

All linked microsatellites mapped consistently on the same linkage groups in the two parental maps and 82% of the markers mapped with the same order along the homologous parental linkage groups with most order changes concentrated on linkage groups 1, 8, 9 and 10 (Figure 2). Although biological reasons such as chromosomal rearrangements between the two species could be involved, these order changes are most likely attributable to analytical causes. These include scoring errors due to allele drop-outs generating apparent recombination events and artifacts of the consensus mapping algorithm when attempting to define order between markers segregating only from one or the other parent with fully informative ones, leading to local reordering of adjacent markers.

Considering a total of 241 microsatellite markers amplified for *E. grandis *and *E. urophylla*, only 12 were expected to be distorted by chance at p ≤ 0.05. However, 29 (12%) were identified but only one would remain significantly distorted after applying a stringent Bonferroni correction. All distorted markers but one were mapped on the consensus map and more than half clustered mainly into two linkage groups (group 2 and 8) with the others scattered across eight linkage groups (Figure 2). The detection of segregation distortion at greater levels than expected by chance has been the rule in mapping reports for many species of plants (e.g. [58,59]). In *Eucalyptus*, essentially all the mapping reports to date detected significant deviation of the expected proportion of distorted markers although at different levels, usually higher in inter specific [4,42,43,45] when compared to intra specific crosses [9,17]. Distorted markers usually cluster in specific regions of the genome therefore excluding genotyping errors as a potential cause. Several post-zygotic selection phenomena could be causing such segregation distortions. However in highly heterogeneous undomesticated forest trees such as *Eucalyptus*, the most likely cause involves the expression of deleterious alleles in heterozygous condition [60] or hybrid incompatibility when crossing divergent species. Myburg *et al*. [61] observed high levels of distortion in backcross families of a *E. grandis *× *E. globulus *F1 hybrid, and used this information to perform a whole-genome analysis of post zygotic barriers between these two species. Although it was possible to demonstrate that positive and negative heterospecific interactions affect introgression rates in such a wide interspecific pedigree, the fact that the study was carried out with dominant AFLP markers precluded a more detailed analysis of the sources of distortion. As properly pointed out in that study, the availability of a large set of microsatellites as described in this report, will be a powerful tool to further investigate the nature of post-zygotic barriers in *Eucalyptus *and thus guide advanced generation hybrid breeding, an exceptionally powerful approach that has been commonly used in *Eucalyptus *to derive elite clones.

The consensus map reported in this work does not contain the RAPD markers originally used to bridge the microsatellites mapped earlier [12,15]. Although the RAPD markers could have been integrated, trying to pack in a very large number of markers could lead to a reduced likelihood support for marker order of the microsatellites, main focus of this study. Furthermore, given the very limited or nil transferability of RAPD to other pedigrees, their presence would add little if any information for future QTL mapping studies. It is important to note, however, that high throughput, high multiplex typing methods such as RAPD and particularly AFLP, will continue to be important complementary tools in *Eucalyptus *genetics for high density mapping [45] and high resolution positioning of disease resistance loci (e.g. [39]) to eventually allow map-based cloning efforts. Novel ultra-high throughput genotyping methods of transferable, sequence specific markers such as DArT, successfully evaluated for *Eucalyptus *[62], and SNP arrays [63], combined with the framework of microsatellites described in this work will most likely provide a robust platform for integrative high-density QTL mapping in the genus *Eucalyptus*.

### Genome length and map coverage

Published estimates of genome length for species of *Eucalyptus *have varied between 919 cM and 1551 cM although most estimates to date have remained around 1300 to 1500 cM (reviewed in [17]), based mostly on markers generated with arbitrary sequence primers. In this work, based only on microsatellites, we obtained a total genome length of 1,814.5 for the female *E. grandis *map, 1,133.4 for the *E. urophylla *male map and 1,567.7 cM for the consensus map. While the length for the *E. urophylla *and consensus maps agree with most estimates to date, the *E. grandis *total length is larger. This observed length is probably inflated by a few markers that, although grouping at LOD >3.0, extend the map in a disproportionate way. They do not map or map in a different order on the consensus map and should therefore be viewed with caution. They were, however included on the maps to provide their preliminary linkage group assignment. These markers are: EMBRA114 on group 3 extending the map in 54 cM; EMBRA88 on group 8 extending the map in 37.7 cM; EMBRA140 on group 9 extending the map in 57 cM. EMBRA102 and EMBRA40 on group 10 extending the map in over 100 cM and displaying map order on the *E. urophylla *on the consensus map. By removing these five markers from the map we arrive to a more conservative total length of 1,562.3 cM. As a reference we compared these observed lengths with the ones obtained earlier for these same parental trees based on 240 and 251 RAPD markers [4]. For *E. grandis*, the conservative estimate of 1,562.3 cM is close to the 1,552 cM of the RAPD map and for *E. urophylla *the microsatellite map with 1,133.4 is also close to the 1,101 cM of the RAPD map. No framework mapping adjustment was carried out in this study as the main objective was to provide the most likely map position for all microsatellite markers reported. However, using the estimated total map length based on the RAPD framework maps (1,620 for *E. grandis *and 1,156 for *E. urophylla*, [4]) the microsatellite parental maps reported in this study cover respectively 96.4% and 98% of the estimated genome length. We were however interested in estimating genome coverage for the consensus map. Using a conservative estimate of the proportion of microsatellite markers that would map as framework markers (i.e. with log likelihood support for order of at least 3.0) we estimated an observed genome coverage of 93%, and a theoretical expected genome coverage of 88.6%. These estimates allow us to propose that the microsatellite consensus map covers approximately 90% of the genome.

### Consolidation of linkage data and comparative mapping in *Eucalyptus*

Using EMBRA markers that were mapped in other independent studies, it was possible to assign other 41 microsatellites to this consensus map at the linkage group level. The definition of the exact order of these 41 microsatelites relative to the other markers on the linkage groups will require genotyping them on this same set of progeny individuals. However the consolidation of linkage data carried out in this study demonstrates the power that a more comprehensive map of microsatellites provides for expanding the opportunities of comparative mapping across *Eucalyptus *species.

Linkage group numbering adopted for this map follows the one originally established for RAPD marker maps. This was an arbitrary numbering that was nevertheless kept to allow integration of microsatellites on the existing maps. Other reports where RFLP, AFLP, EST, other microsatellites and candidate genes were mapped have used different numbering. There is clearly a need to unify linkage group numbering for *Eucalyptus *species to facilitate the continued addition of new markers and genes. While the numbering proposed here now makes a first step toward this direction, the establishment of a correct numbering system for the chromosomes and hence for the linkage groups, should derive from cytogenetic studies using previously screened BAC with specific microsatellites as *in situ *hybridization probes.

This consolidation of microsatellite linkage mapping data will also expand the prospects of making comparative analysis of putative QTL synteny such as that carried out in *Eucalyptus *by Marques *et al*. [30] for vegetative propagation traits and by Thamarus *et al*. [38] for wood density QTLs. Another interesting opportunity is the proposition of putative candidate genes for major effect QTLs. For example, an early flowering QTL named *Eef1 *was recently mapped by Missiaggia *et al*. [46] on linkage group 2 flanked by markers EMBRA27 and EMBRA164. Linkage group 2 corresponds to linkage group 4 of Thamarus *et al*. [17] where EAP1, the *Eucalyptus *functional equivalent of the *Arabidopsis Apetala1 *gene was mapped. As the ectopic expression of the EAP1 in *Arabidopsis *driven by the 35S promoter caused plants to flower earlier [64] this comparative mapping information provides an interesting lead to test this gene as a candidate underlying the *Eef1 *QTL. In a similar way, the linkage group assignment of the CCR gene at the tip of linkage group 10 of Thamarus *et al*. [17] indicates that this candidate gene should be located at one of the tips of linkage group 10 of this consensus map, either close to EMBRA33 and EMBRA10 or at the other end close to EMBRA155 and EMBRA127. Polymorphisms at the CCR gene have recently been associated with variation in microfibril angle in *Eucalyptus nitens *and *E. globulus *[65]. This combined information can be very valuable for a directed screening of microsatellite markers linked to CCR in populations segregating for microfibril angle in an attempt to validate this QTL in different populations or *Eucalyptus *species. In an analogous way, once microsatellites are mapped close to the EgMYB2 gene, recently shown to co-localize with a QTL for lignin content [66], it will be possible to validate this QTL and evaluate the effect of this candidate gene in variable genetic backgrounds.

## Conclusion

This report describes the development of a novel set of 230 EMBRA microsatellites, the construction of the first consensus linkage map with 234 mapped loci, covering ~90% of the *Eucalyptus *genome. Based on a set of shared microsatellites with other *Eucalyptus *mapping studies, a set of other 41 microsatellites, candidate genes and QTLs for wood and flowering traits were assigned to the consensus map making it the first consolidated source of existing linkage information for species of *Eucalyptus*. This report significantly increases the availability of microsatellite markers for species of *Eucalyptus*, corroborating the high conservation of microsatellite flanking sequences and locus ordering across species of the genus. This work represents an important step forward for *Eucalyptus *comparative genomics, opening stimulating perspectives for evolutionary studies and molecular breeding applications in species of the genus. The availability of microsatellites for *Eucalyptus *should undergo a quick expansion in the next few years based on the large EST and genomic sequence databases available. The generalized use of increasingly larger sets of interspecific transferable markers and consensus mapping information will allow faster and more detailed investigations of QTL synteny among species, validation of expression-QTL across variable genetic backgrounds and positioning of a growing number of candidate genes to be tested in association mapping experiments.

## Methods

### Plant material and DNA extraction

Genetic mapping of the new set of microsatellite markers was performed on a mapping population of 92 F1 individuals derived from a cross between a female *E. grandis *(clone G44) and male *E. urophylla *(clone U28) [4]. Both species belong to the same subgenus *Symphyomyrtus*. For the characterization of the mapping information content, 32 individual trees of *Eucalyptus*, 16 from *E. grandis *and 16 from *E. urophylla*, were randomly chosen from a germplasm collection composed of trees grown from seeds collected in natural populations. Genomic DNA from leaves stored at -20°C was extracted as described earlier [4].

### Microsatellite marker analysis

The development, PCR amplification, detection, inheritance and segregation analysis of the microsatellites were carried out as described previously [12]. In this work, three hundred and eighty new microsatellite primer pairs were designed and tested for amplification and polymorphism using the two parents and a progeny sample of four individuals. The PCR amplification conditions were: 5 min at 94°C, 30 cycles of 1 min at 94°C, 1 min at the primer specific annealing temperature, 1 min at 72°C, and 5 min at 72°C for final extension. Different annealing temperatures, ranging from 54°C to 60°C, were used to amplify specific microsatellite markers. The microsatellite markers were identified by the same acronym (EMBRA, *Eucalyptus *Microsatellite from Brazil) adopted earlier [12], followed by a sequentially assigned number.

### Parental map linkage analysis

Separate linkage maps for each parent tree were constructed based exclusively on microsatellites that were in heterozygous state and thus segregating in the expected 1:1 ratio. Seventy microsatellites published previously (EMBRA1 through EMBRA70) [12,15] were mapped together with the novel set of segregating markers developed in this study. Linkage analyses were performed using MapMaker 2.0 [67] for Macintosh. Linked markers were first placed into linkage groups using the "group" command with a threshold LOD score of 3.0 and a maximum recombination fraction (theta) of 0.35. The "first-order" and "compare" commands were then used to identify the most probable marker order within a linkage group. The "ripple" command was used to verify the log likelihood support for local order. For final marker ordering we chose not to adopt a stringent log likelihood support of 3.0 followed by keeping only framework markers, as the main objective of the study was to position all the developed microsatellites on the linkage map. The log-likelihood support for marker ordering was therefore variable in different map segments. The marker orders presented were the best possible orders based on the highest overall likelihood when all markers were included in the analysis. Recombination fractions were transformed to estimated map distances by the Kosambi map function. Linkage group numbering followed the one proposed originally by Grattapaglia and Sederoff [4] for the RAPD genetic map of *E. grandis *and later adopted for microsatellite mapping [12].

### Consensus map construction and genome coverage

A combined data set with the gametic classes of all 92 F1 progeny was created to construct an integrated linkage map. As a result of using outbred parents, two distinct types of segregation were found: less informative 1:1 segregating loci, with two different alleles segregating from only one of the two parents (pseudo-testcross), and a fully informative 1:1:1:1 segregating locus with both parents heterozygous and three or four different alleles segregating from the two parents. A chi-square test was performed (alpha = 0.05) to test for deviation of genotypic classes from the expected Mendelian inheritance ratios. The integrated linkage analysis, including estimation of recombination fraction and locus order was performed using the windows-based package OUTMAP [68] specifically developed to map codominant loci in outcrossed trees with higher efficiency when compared to other existing programs, Map figures were constructed using the computer program QGene [69]. Fully informative markers segregating 1:1:1:1 were used for a comparative mapping analysis to reveal discrepancies in the microsatellite marker orders between the two independent parental maps and between them and the consensus map.

The estimated genome length (G_est_) of the consensus map was estimated with the commonly used Hulbert estimate [70]. For generating this estimate, only framework markers have to be considered to avoid an overestimation of genome size due to clustered markers. We used a conservative estimate of 53% of the consensus mapped markers being framework markers, i.e. assigned with LOD support of 3.0 or more, based on the proportion of framework markers estimated earlier for these two parental maps [4]. The observed genome coverage (C_obs_) was then estimated as the ratio between the total observed genome length (G_obs_) to the estimated genome length (G_est_). A theoretical expected genome coverage (C_exp_) for the consensus map was also estimated using the equation C_exp _= 1-e^-XN/1.25Gest ^[71], where X is the maximum distance between two adjacent markers, N is the number of framework markers and (G_est_) is the estimated genome length.

### Microsatellite characterization

Allelic diversity and estimates of observed and expected heterozygosity of thirty-five selected microsatellites were obtained as described previously [12] after genotyping a panel of 32 unrelated trees, 16 of *E. grandis *and 16 of *E. urophylla *sampled from natural populations. These 35 loci were selected to be recommended as anchor loci based on their uniform distribution along the eleven linkage groups, the generation of easily interpretable genotypes and high transferability to other *Eucalyptus *species. The following parameters were determined: the total number of alleles observed in each species and shared between them, the observed (H_obs_) and expected (H_exp_) heterozygosity for each species and combined.

### Compilation of microsatellite linkage information

A compilation of all microsatellite linkage information available in the literature to date was carried out to establish homology between the linkage groups of this consensus map and other maps published for *E. grandis *× *E. urophylla *[72] and other *Eucalyptus *species [17,30,44]. EMBRA markers localized on all these maps were used as anchors to define the number correspondence between linkage groups. These anchor markers allowed assigning microsatellites developed in other laboratories (acronyms EMCRC, Eg, En, Es) as well as candidate genes mapped by RFLP, to linkage groups in this consensus map.

## Authors' contributions

RPVB carried out most of the experimental work, including microsatellite development, genotyping and data analysis, parental map construction and drafting of the first version of the manuscript. ERW was responsible for constructing the first version of the consensus map. CB contributed to the development of microsatellites. DG was responsible for project conception, data analysis, compilation of existing linkage information from the literature and writing the final version of the manuscript.

## Supplementary Material

Additional file 1 Consolidated list of 300 EMBRA microsatellite markers. Summary of all the information for the 230 new microsatellite markers reported in this study (EMBRA71 through EMBRA395) plus 70 published earlier (EMBRA1 through EMBRA70) [10,26] including the Genbank accession number of the original sequences from which the microsatellite primer pairs were designed. Information includes: the microsatellite EMBRA number, repeat motif, forward and reverse primer sequences, recommended annealing temperature (°C), size of expected product in basepairs, and linkage group assignment. Map position is described for 161 plus 4 duplicated loci, (see text), totalling 165 loci that were mapped in the *E. grandis *G44 × *E. urophylla *U28 mapping population. (n.s. = microsatellite not segregating in the mapping pedigree; n.a. = not available raw sequence data for submisison to Genbank). Click here for file

Additional file 2 Genetic characterization of anchor microsatellite markers. Summary information on the genetic diversity of 35 microsatellites selected as anchor loci as evaluated on a panel of 32 unrelated individual trees of *Eucalyptus *sp. (H_exp _= expected heterozigosity; H_obs _= observed heterozigosity). Click here for file

## Figures and Tables

**Table 1:** Comparative summary and main attributes of the parental and consensus maps of *Eucalyptus*.

		***E. grandis *map**						***E. urophylla *map**						**Consensus map**						
Linkage group	Total # markers mapped	# markers mapped on the *E. grandis *map	# mapped markers segregating only from *E. grandis*	Size in cM	Mean distance between markers, cM)^b^	# markers mapped on the *E. grandis *and on consensus map	# markers mapped only on *E. grandis *map but not on consensus map	# markers mapped on the *E. urophylla *map	# mapped markers segregating only from *E. urophylla*	Size in cM	Mean distance between markers, cM)^b^	# markers mapped on the *E. urophylla *and on consensus map	# markers mapped only on *E. urophylla *map but not on consensus map	# fully informative markers on consensus map	# markers on consensus map segregating only in *E. grandis*	# markers on consensus map segregating only in *E. urophylla*	# markers mapped on consensus map	Size in cM	Mean distance between markers, cM)^b^	# markers mapped on *E. grandis *and *E urophylla *but not on consensus
1	22	21	6	164.2	15.9	18	2	16	1	134.3	9.0	14	1	14	4	0	18	151.0	8.9	1
2	28	24	7	169.7	7.4	17	4	21	4	97.2	4.9	18	0	14	3	4	21	144.0	7.2	3
3	15	14	4	143.2	11.0	11	3	11	1	60.7	6.1	11	0	10	1	1	12	73.5	6.7	0
4	19	13	3	123.6	10.3	13	0	16^a^	6	92.9	6.6	16	0	10	3	6	19	119.7	6.7	0
5	26	24	6	202.1	8.8	23	0	20	2	134.9	7.1	19	0	17	6	2	25	168.7	7.0	1
6	21	20	18	164.8	8.7	14	6	3	1	50.9	25.5	2	1	2	12	0	14	122.1	9.4	0
7	24	20	6	100.2	5.3	19	1	18	4	82.2	4.8	18	0	14	5	4	23	95.4	4.3	0
8	28	27	8	274.1	10.5	24	3	20	1	141.0	7.4	20	0	19	5	1	25	211.8	8.8	0
9	20	16	4	202.6	13.5	14	2	16	4	139.4	9.3	16	0	12	2	4	18	206.9	12.2	0
10	18	11	2	184.5	18.5	8	2	16	7	183.0	12.2	15	0	8	0	7	15	209.0	14.9	1
11	13	12	10	85.5	7.8	11	1	3	1	16.9	8.5	3	0	2	9	1	12	65.6	6.0	0

**TOTAL**	**234**	**202**	**74**	**1,814.5**	**10.7^c^**	**172**	**24**	**160**	**32**	**1,133.4**	**9.2^c^**	**152**	**2**	**122**	**50**	**30**	**202**	**1,567.7**	**8.4^c^**	**6**

^a ^Linkage group # 4 in *E. urophylla *is composed by two separate linkage groups.

^b ^Mean distance between markers is computed as the arithmetic mean of the map distances between adjacent markers shown in Figure 2.

^c ^Arithmetic mean of the mean distance between markers in each linkage group.

**Table 2:** Microsatellite markers that displayed null alleles, indicated by (-). and their respective mating configuration observed.

**Microsatellite**	***E. grandis***	***E. urophylla***	**Linkage group**
EMBRA84	1/2	-/-	1
EMBRA21	1/-	-/3	10
EMBRA43	1/2	-/-	2
EMBRA55	1/2	3/-	2
EMBRA111	1/-	3/4	5
EMBRA118	1/2	-/-	8
EMBRA129	1/2	-/-	6
EMBRA147	1/2	3/-	1
EMBRA153	1/2	-/-	10
EMBRA159	1/2	-/-	2
EMBRA185	1/2	-/-	2
EMBRA187	1/-	-/-	6
EMBRA199	1/2	-/-	1
EMBRA207	1/-	-/-	2
EMBRA216	1/-	3/-	2
EMBRA218a	1/2	3/-	2
EMBRA224	1/2	-/-	8
EMBRA230	1/2	-/-	6
EMBRA236	1/2	-/-	11
EMBRA361	1/2	-/-	3

**Table 3:** Homologies of linkage groups among different *Eucalyptus sp*. mapping studies that employed microsatellite markers. Localization of microsatellites derived and mapped from other *Eucalyptus *species [17, 44] and candidate genes [17], on the linkage groups of the consensus map of *E. grandis *and *E. urophylla*. The EMBRA microsatellites that allowed this analysis are listed in italics between parenthesis.

Linkage groups (this study)	Linkage groups Thamarus *et al*. [17]	CSIRO Microsatellites Thamarus *et al*. [17]	Candidate genes Thamarus *et al*. [17]	Linkage groups Bundock *et al*. [44]	CRC Microsatellites Bundock *et al*. [44]
1	7	En006, Eg084, Eg065, Eg134, (*EMBRA6*)	COMT, SAMS (est), Laccase (est)	-	-
2	4^a^	Eg008, Eg076, Eg111, Eg086, Eg096	EAP1, PAL	-	-
3	8^b^	Eg094, Eg098, Eg131	ECS1	-	-
4	11^g^	Eg128, En011, Eg030	MsaS2	-	-
5	9	(*EMBRA5*)	AGE2	3	EMCRC4, (*EMBRA5*), (*EMBRA9*)
6	6^c^	Eg015b, Eg062, En016,	EXS2	-	-
7	2^d^	Eg061, En012, En014	CCOAOMT, ECA1, XET(est)	2	EMCRC9, (*EMBRA7*)
8	1^e^	Es076, En013, En015, Eg117, Eg126, En010		6	EMCRC8, (*EMBRA3*)
9	5	Eg067, Eg089, Eg015a, Es140, Eg115	EXS2,	1	EMCRC1, (*EMBRA17*, *EMBRA18*)
10	10^f^	Eg023, Eg091, Es115	CCR	-	-
11	3	Eg099, Eg024, (*EMBRA2*)	4CL, AGE1, ELF, SAMS (est)	-	-

^a ^Homology was established based on markers Eg086 and Eg096 mapped by Missiaggia [72]

^b ^Homology was established based on markers Eg094 and Eg098 mapped by Missiaggia [72]

^c ^Homology was established based on marker Eg062 mapped by Missiaggia [72]

^d ^Homology was established based on markers En014 mapped by Missiaggia [72]

^e ^Homology was established based on markers En010 and Eg126 mapped by Missiaggia [72]

^f ^Homology was established based on markers Eg091 mapped by Missiaggia [72]

^g ^Homology was established based on the fact that only linkage group 4 of this work and group 11 of Thamarus *et al*. [17] were left.

## References

[B1] FAO (2000). Global forest resources assessment 2000 - Main report..

[B2] Eldridge K, Davidson J, Harwood C, van Wyk G (1993). Eucalypt domestication and breeding.

[B3] Ritter E, Gebhardt C, Salamini F (1990). Estimation of recombination frequencies and construction of RFLP linkage maps in plants from crosses between heterozygous parents. Genetics.

[B4] Grattapaglia D, Sederoff RR (1994). Genetic linkage maps of Eucalyptus grandis and Eucalyptus urophylla using a pseudo-testcross: mapping strategy and RAPD markers. Genetics.

[B5] Grattapaglia D, Wilcox P, Chaparro JX, O'Malley DM, McCord S, Whetten RW, McIntyre L, Sederoff RR (1991). A RAPD map of loblolly pine in 60 days.. Third International Congress of the International Society for Plant Molecular Biology.

[B6] Carlson JE, Tulsieram LK, Glaubitz JC, Luk VWK, Kauffeldt C, Rutledge R (1991). Segregation of random amplified DNA markers in F1 progeny of conifers. Theor Appl Genet.

[B7] Tulsieram LK, Glaubitz JC, Kiss G, Carlson JE (1992). Single tree genetic linkage mapping in conifers using haploid DNA from megagametophytes. Biotechnology (N Y).

[B8] Devey ME, Fiddler TA, Liu BH, Knapp SJ, Neale DB (1994). An RFLP linkage map for loblolly pine based on a three-generation outbred pedigree. Theor Appl Genet.

[B9] Byrne M, Murrell JC, Allen B, Moran GF (1995). An integrated genetic linkage map for eucalypts using RFLP, RAPD and isozyme markers. Theor Appl Genet.

[B10] Komulainen P, Brown GR, Mikkonen M, Karhu A, Garcia-Gil MR, O'Malley D, Lee B, Neale DB, Savolainen O (2003). Comparing EST-based genetic maps between Pinus sylvestris and Pinus taeda. Theor Appl Genet.

[B11] Krutovsky KV, Troggio M, Brown GR, Jermstad KD, Neale DB (2004). Comparative mapping in the Pinaceae. Genetics.

[B12] Brondani RPV, Brondani C, Tarchini R, Grattapaglia D (1998). Development, characterization and mapping of microsatellite markers in Eucalyptus grandis and E. urophylla.. Theor Appl Genet.

[B13] Grattapaglia D, Jain SM and Minocha SC (2000). Molecular breeding of Eucalyptus - State of the art, operational applications and technical challenges. Molecular biology of woody plants, Forestry Sciences, Volume 64.

[B14] Gion JM, Rech P, Grima-Pettenati J, Verhaegen D, Plomion C (2000). Mapping candidate genes in Eucalyptus with emphasis on lignification genes.. Mol Breeding.

[B15] Brondani RPV, Brondani C, Grattapaglia D (2002). Towards a genus-wide reference linkage map for Eucalyptus based exclusively on highly informative microsatellite markers. Mol Genet Genomics.

[B16] Barreneche T, Bodenes C, Lexer C, Trontin JF, Fluch S, Streiff R, Plomion C, Roussel G, Steinkellner H, Burg K, Favre JM, Glossl J, Kremer A (1998). A genetic linkage map of Quercus robur L. (pedunculate oak) based on RAPD, SCAR, microsatellite, minisatellite, isozyme and 5S rDNA markers. Theor Appl Genet.

[B17] Thamarus K, Groom K, Murrell J, Byrne M, Moran G (2002). A genetic linkage map for Eucalyptus globulus with candidate loci for wood, fibre and floral traits. Theor Appl Genet.

[B18] Tani N, Takahashi T, Iwata H, Mukai Y, Ujino-Ihara T, Matsumoto A, Yoshimura K, Yoshimaru H, Murai M, Nagasaka K, Tsumura Y (2003). A consensus linkage map for sugi (Cryptomeria japonica) from two pedigrees, based on microsatellites and expressed sequence tags. Genetics.

[B19] Achere V, Faivre-Rampant P, Jeandroz S, Besnard G, Markussen T, Aragones A, Fladung M, Ritter E, Favre JM (2004). A full saturated linkage map of Picea abies including AFLP, SSR, ESTP, 5S rDNA and morphological markers. Theor Appl Genet.

[B20] Cervera MT, Storme V, Ivens B, Gusmao J, Liu BH, Hostyn V, Van Slycken J, Van Montagu M, Boerjan W (2001). Dense genetic linkage maps of three Populus species (Populus deltoides, P. nigra and P. trichocarpa) based on AFLP and microsatellite markers. Genetics.

[B21] Zhou Y, Gwaze DP, Reyes-Valdes MH, Bui T, Williams CG (2003). No clustering for linkage map based on low-copy and undermethylated microsatellites. Genome.

[B22] Yin TM, DiFazio SP, Gunter LE, Riemenschneider D, Tuskan GA (2004). Large-scale heterospecific segregation distortion in Populus revealed by a dense genetic map. Theor Appl Genet.

[B23] Steane DA, Vaillancourt RE, Russell J, Powell W, Marshall D, Potts BM (2001). Development and characterisation of microsatellite loci in Eucalyptus globulus (Myrtaceae). Silvae Genetica.

[B24] Byrne M, Marquez-Garcia MI, Uren T, Smith DS, Moran GF (1996). Conservation and genetic diversity of microsatellite loci in the genus Eucalyptus.. Aust J Botany.

[B25] Glaubitz JC, Emebiri LC, Moran GF (2001). Dinucleotide microsatellites from Eucalyptus sieberi: inheritance, diversity, and improved scoring of single-base differences. Genome.

[B26] Ottewell KM, Donnellan SC, Moran GF, Paton DC (2005). Multiplexed microsatellite markers for the genetic analysis of Eucalyptus leucoxylon (Myrtaceae) and their utility for ecological and breeding studies in other eucalyptus species. J Hered.

[B27] CSIRO (2000). http://www.ffp.csiro.au/tigr/molecular/eucmsps.html.

[B28] Steane DA, Jones RC, Vaillancourt RE (2005). A set of chloroplast microsatellite primers for Eucalyptus (Myrtaceae). Mol Ecol Notes.

[B29] Kirst M, Brondani RPV, Brondani C, Grattapaglia D, IUFRO  (1997). Screening of designed primer pairs for recovery of microsatellite markers and their transferability among species of Eucalyptus: ; IUFRO Conference on Eucalyptus Genetics and Silviculture..

[B30] Marques M, Brondani RPV, Grattapaglia D, Sederoff R (2002). Conservation and synteny of SSR loci and QTLs for vegetative propagation in four Eucalyptus species. Theor Appl Genet.

[B31] Grattapaglia D, Bertolucci FL, Sederoff RR (1995). Genetic mapping of QTLs controlling vegetative propagation in Eucalyptus grandis and E. urophylla using a pseudo-testcross strategy and RAPD markers. Theor Appl Genet.

[B32] Grattapaglia D, Bertolucci FL, Penchel R, Sederoff RR (1996). Genetic mapping of quantitative trait loci controlling growth and wood quality traits in Eucalyptus grandis using a maternal half-sib family and RAPD markers. Genetics.

[B33] Byrne M, Murrell JC, Owen JV, Kriedemann P, Williams ER, Moran GF (1997). Identification and mode of action of quantitative trait loci affecting seedling height and leaf area in Eucalyptus nitens.. Theor Appl Genet.

[B34] Verhaegen D, Plomion C, Gion JM, Poitel M, Costa P, Kremer A (1997). Quantitative trait dissection analysis in Eucalyptus using RAPD markers, 1. Detection of QTL in interspecific hybrid progeny, stability of QTL expression across different ages. Theor Appl Genet.

[B35] Shepherd M, Chaparro JX, Teasdale R (1999). Genetic mapping of monoterpene composition in an interspecific eucalypt hybrid. Theor Appl Genet.

[B36] Marques CM, Vasquez-Kool J, Carocha VJ, Ferreira JG, O'Malley DM, Liu BH, Sederoff R (1999). Genetic dissection of vegetative propagation traits in Eucalyptus tereticornis and E. globulus.. Theor Appl Genet.

[B37] Myburg AA (2001). Genetic architecture of hybrid fitness and wood quality traits in a wide interspecific cross of Eucalyptus tree species.

[B38] Thamarus K, Groom K, Bradley A, Raymond CA, Schimleck LR, Williams ER, Moran GF (2004). Identification of quantitative trait loci for wood and fibre properties in two full-sib properties of Eucalyptus globulus. Theor Appl Genet.

[B39] Junghans DT, Alfenas AC, Brommonschenkel SH, Oda S, Mello EJ, Grattapaglia D (2003). Resistance to rust ( Puccinia psidii Winter) in eucalyptus: mode of inheritance and mapping of a major gene with RAPD markers. Theor Appl Genet.

[B40] Kirst M, Myburg AA, De Leon JP, Kirst ME, Scott J, Sederoff R (2004). Coordinated genetic regulation of growth and lignin revealed by quantitative trait locus analysis of cDNA microarray data in an interspecific backcross of Eucalyptus. Plant Physiol.

[B41] Kirst M, Basten CJ, Myburg AA, Zeng ZB, Sederoff RR (2005). Genetic architecture of transcript-level variation in differentiating xylem of a eucalyptus hybrid. Genetics.

[B42] Verhaegen D, Plomion C (1996). Genetic mapping in Eucalyptus urophylla and Eucalyptus grandis using RAPD markers. Genome.

[B43] Marques CM, Araujo JA, Ferreira JG, Whetten R, O'Malley DM, Liu BH, Sederoff R (1998). AFLP genetic maps of Eucalyptus globulus and E. tereticornis. Theor Appl Genet.

[B44] Bundock PC, Hayden M, Vaillancourt RE (2000). Linkage maps of Eucalyptus globulus using RAPD and microsatellite markers. Silvae Genetica.

[B45] Myburg AA, Griffin AR, Sederoff RR, Whetten RW (2003). Comparative genetic linkage maps of Eucalyptus grandis, Eucalyptus globulus and their F1 hybrid based on a double pseudo-backcross mapping approach. Theor Appl Genet.

[B46] Missiaggia AA, Piacezzi A, Grattapaglia D (2005). Genetic mapping of Eef1, a major effect QTL for early flowering in Eucalyptus grandis. Tree Genet Genomes.

[B47] Agrama HA, George TL, Salah SF (2002). Construction of genome map for Eucalyptus camaldulensis DEHN.. Silvae Genetica.

[B48] Lourenço RT (2004). Sample sequencing of 3 Megabases of shotgun DNA of Eucalyptus grandis: genome structure, repetitive elements and genes..

[B49] Grattapaglia D, Alfenas AC, Coelho ASG, Bearzoti E, Pappas GJ, Pasquali G, Pereira G, Colodette J, Gomide JL, Bueno J, Cascardo JC, Brondan RPV, Brommonschenkel SH (2004). Building resources for molecular breeding of Eucalyptus: ; International IUFRO Conference - Eucalyptus in a changing world, Aveiro Portugal..

[B50] Morgante M, Hanafey M, Powell W (2002). Microsatellites are preferentially associated with nonrepetitive DNA in plant genomes. Nat Genet.

[B51] Groover A, Devey M, Fiddler T, Lee J, Megraw R, Mitchel-Olds T, Sherman B, Vujcic S, Williams C, Neale D (1994). Identification of quantitative trait loci influencing wood specific gravity in an outbred pedigree of loblolly pine. Genetics.

[B52] Dakin EE, Avise JC (2004). Microsatellite null alleles in parentage analysis. Heredity.

[B53] Provan J, Powell W, Waugh R (1996). Microsatellite analysis of relationships within cultivated potato (Solanum tuberosum). Theor Appl Genet.

[B54] Peakall R, Gilmore S, Keys W, Morgante M, Rafalski A (1998). Cross-species amplification of soybean (Glycine max) simple sequence repeats (SSRs) within the genus and other legume genera: implications for the transferability of SSRs in plants. Mol Biol Evol.

[B55] Barreneche T, Casasoli M, Russell K, Akkak A, Meddour H, Plomion C, Villani F, Kremer A (2004). Comparative mapping between Quercus and Castanea using simple-sequence repeats (SSRs). Theor Appl Genet.

[B56] Maliepaard C, Alston FH, van Arkel G, Brown LM, Chevreau E, Dunemann F, Evans KM, Gardiner S, Guilford P, van Heusden AW, Janse J, Laurens F, Lynn JR, Manganaris AG, den Nijs APM, Periam N, Rikkerink E, Roche P, Ryder C, Sansavini S, Schmidt H, Tartarini S, Verhaegh JJ, Vrielink-van Ginkel M, King GJ (1998). Aligning male and female linkage maps of apple (Malus pumila Mill.) using multi-allelic markers. Theor Appl Genet.

[B57] Lespinasse D, Rodier-Goud M, Grivet L, Leconte A, Legnate H, Seguin M (2000). A saturated genetic linkage map of rubber tree Hevea spp. based on RFLP, AFLP, microsatellite, and isozyme markers. Theor Appl Genet.

[B58] Xu Y, Zhu L, Xiao J, Huang N, McCouch SR (1997). Chromosomal regions associated with segregation distortion of molecular markers in F2, backcross, doubled haploid, and recombinant inbred populations in rice (Oryza sativa L.). Mol Genet Genomics.

[B59] Bradshaw HD, Stettler RF (1994). Molecular genetics of growth and development in Populus II. Segregation distortion due to genetic load. Theor Appl Genet.

[B60] Potts BM, Wiltshire RJE, Wiliams J and Woinarski J (1997). Eucalypt genetics and genecology. Eucalypt Ecology: Individuals to Ecosystems.

[B61] Myburg AA, Vogl C, Griffin AR, Sederoff RR, Whetten RW (2004). Genetics of postzygotic isolation in Eucalyptus: whole-genome analysis of barriers to introgression in a wide interspecific cross of Eucalyptus grandis and E. globulus. Genetics.

[B62] Lezar S, Myburg AA, Berger DK, Wingfield MJ, Wingfield BD (2004). Development and assessment of microarray-based DNA fingerprinting in Eucalyptus grandis. Theor Appl Genet.

[B63] Borevitz JO, Liang D, Plouffe D, Chang HS, Zhu T, Weigel D, Berry CC, Winzeler E, Chory J (2003). Large-scale identification of single-feature polymorphisms in complex genomes. Genome Res.

[B64] Kyozuka J, Harcourt R, Peacock WJ, Dennis ES (1997). Eucalyptus has functional equivalents of the Arabidopsis AP1 gene. Plant Mol Biol.

[B65] Thumma BR, Nolan MF, Evans R, Moran GF (2005). Polymorphisms in cinnamoyl CoA reductase (CCR) are associated with variation in microfibril angle in Eucalyptus spp. Genetics.

[B66] Goicoechea M, Lacombe E, Legay S, Mihaljevic S, Rech P, Jauneau A, Lapierre C, Pollet B, Verhaegen D, Chaubet-Gigot N, Grima-Pettenati J (2005). EgMYB2, a new transcriptional activator from Eucalyptus xylem, regulates secondary cell wall formation and lignin biosynthesis. Plant J.

[B67] Lander ES, Green P, Abrahamson J, Barlow A, Daly MJ, Lincoln SE, Newburg L (1987). MAPMAKER: an interactive computer package for constructing primary genetic linkage maps of experimental and natural populations. Genomics.

[B68] Butcher A, Williams ER, Whitaker D, Ling S, Speed P, Moran F (2002). Improving linkage analysis in outcrossed forest trees - an example from Acacia mangium. Theor Appl Genet.

[B69] Nelson JC (1997). QGENE: software for marker-based genomic analysis and breeding. Mol Breeding.

[B70] Hulbert SH, Ilott TW, Legg EJ, Lincoln SE, Lander ES, Michelmore RW (1988). Genetic analysis of the fungus, Bremia lactucae, using restriction fragment length polymorphisms. Genetics.

[B71] Lange KBM (1982). How many polymorphic genes will it take to span the human genome?. Am J Hum Genet.

[B72] Missiaggia AA (2005). Mapeamento genético de QTL para qualidade da madeira e florescimento precoce e estudos de expressão gênica alelo específica em Eucalyptus spp..

