# Supplementary material for: A microsatellite-based consensus linkage map for species of Eucalyptus and a novel set of 230 microsatellite markers for the genus
Source: BMC Plant Biol. 2006 Sep 22;6:20. doi: 10.1186/1471-2229-6-20 (PMC1599733; doi:10.1186/1471-2229-6-20)
Supplement: Additional file 2 — Genetic characterization of anchor microsatellite markers. Summary information on the genetic diversity of 35 microsatellites selected as anchor loci as evaluated on a panel of 32 unrelated individual trees of Eucalyptus sp. (Hexp = expected heterozigosity; Hobs = observed heterozigosity). [file 1471-2229-6-20-S2.doc]

**Additional file 2**: Genetic characterization of anchor microsatellites.

| Microsatellite | Linkage group | Allele Size range, bp) | # Alleles *E. urophylla* | # Alleles *E. grandis* | # Shared alleles | Total # alleles | H_obs_ .  *E. urophylla* | H_exp_ .  *E. urophylla* | H_obs_ .  *E. grandis* | H_exp_ .  *E. grandis* | H_obs_ combined | H_exp_ combined |
| --- | --- | --- | --- | --- | --- | --- | --- | --- | --- | --- | --- | --- |
| EMBRA5 | 5 | 105 - 140 | 14 | 15 | 11 | 18 | 0.75 | 0.91 | 0.81 | 0.91 | 0.78 | 0.93 |
| EMBRA13 | 9 | 80 - 290 | 10 | 9 | 5 | 14 | 0.71 | 0.85 | 0.47 | 0.85 | 0.59 | 0.91 |
| EMBRA88 | 8 | 105 - 155 | 18 | 13 | 10 | 21 | 1.00 | 0.92 | 0.81 | 0.85 | 0.91 | 0.92 |
| EMBRA91 | 2 | 110 - 180 | 16 | 11 | 7 | 20 | 0.81 | 0.92 | 0.86 | 0.84 | 0.83 | 0.90 |
| EMBRA94 | 6 ^a^ | 210 - 245 | 11 | 9 | 7 | 13 | 0.56 | 0.84 | 0.60 | 0.86 | 0.58 | 0.87 |
| EMBRA100 | 1 | 210 - 260 | 12 | 14 | 9 | 17 | 0.44 | 0.83 | 0.56 | 0.89 | 0.50 | 0.90 |
| EMBRA105 | 6 | 115 - 150 | 14 | 12 | 8 | 18 | 0.75 | 0.88 | 0.87 | 0.84 | 0.81 | 0.89 |
| EMBRA119 | 8 | 180 - 200 | 5 | 8 | 5 | 8 | 0.56 | 0.63 | 0.75 | 0.85 | 0.66 | 0.80 |
| EMBRA121 | 7 | 210 - 250 | 8 | 9 | 8 | 9 | 0.88 | 0.86 | 0.50 | 0.81 | 0.69 | 0.86 |
| EMBRA124 | 10 | 130 - 190 | 14 | 10 | 7 | 17 | 0.44 | 0.90 | 0.44 | 0.87 | 0.44 | 0.92 |
| EMBRA125 | 3 | 170 - 220 | 13 | 14 | 8 | 19 | 0.69 | 0.89 | 0.81 | 0.88 | 0.75 | 0.91 |
| EMBRA126 | 2 | 105 - 145 | 12 | 11 | 9 | 14 | 0.81 | 0.85 | 0.75 | 0.88 | 0.78 | 0.89 |
| EMBRA131 | 9 | 110 - 145 | 8 | 11 | 5 | 14 | 0.88 | 0.79 | 1.00 | 0.85 | 0.94 | 0.87 |
| EMBRA135 | 5 | 100 - 145 | 7 | 9 | 6 | 10 | 0.75 | 0.80 | 0.69 | 0.79 | 0.71 | 0.81 |
| EMBRA146 | 4 | 90 - 140 | 13 | 10 | 8 | 15 | 0.69 | 0.87 | 0.56 | 0.85 | 0.63 | 0.91 |
| EMBRA154b | 6 | 205 - 260 | 10 | 10 | 9 | 11 | 0.56 | 0.88 | 0.56 | 0.85 | 0.56 | 0.88 |
| EMBRA156 | 4 | 125 - 165 | 10 | 10 | 5 | 15 | 0.53 | 0.87 | 0.67 | 0.79 | 0.60 | 0.89 |
| EMBRA167 | 7 | 165 - 210 | 10 | 14 | 9 | 15 | 0.27 | 0.84 | 0.69 | 0.90 | 0.48 | 0.91 |
| EMBRA168 | 5 | 75 - 90 | 5 | 9 | 5 | 9 | 0.63 | 0.71 | 0.81 | 0.84 | 0.72 | 0.81 |
| EMBRA176 | 11 | 115 - 170 | 10 | 12 | 5 | 17 | 0.25 | 0.88 | 0.44 | 0.89 | 0.34 | 0.92 |
| EMBRA184 | 7 | 155 - 240 | 11 | 11 | 9 | 13 | 0.50 | 0.87 | 0.75 | 0.89 | 0.63 | 0.90 |
| EMBRA186 | 4 | 140 - 185 | 8 | 12 | 7 | 13 | 0.53 | 0.75 | 0.81 | 0.88 | 0.68 | 0.89 |
| EMBRA191 | 11 | 155 - 225 | 12 | 11 | 5 | 18 | 0.44 | 0.89 | 0.56 | 0.85 | 0.50 | 0.92 |
| EMBRA200 | 7 | 115 - 190 | 12 | 12 | 8 | 16 | 0.56 | 0.87 | 0.64 | 0.89 | 0.60 | 0.90 |
| EMBRA201 | 2 | 145 - 210 | 16 | 18 | 12 | 22 | 1.00 | 0.92 | 0.88 | 0.93 | 0.94 | 0.95 |
| EMBRA204 | 9 | 118 - 155 | 12 | 15 | 10 | 17 | 0.73 | 0.89 | 0.69 | 0.91 | 0.71 | 0.91 |
| EMBRA210 | 9 | 195 - 215 | 11 | 8 | 6 | 13 | 0.75 | 0.88 | 0.75 | 0.85 | 0.75 | 0.88 |
| EMBRA211 | 9 | 260 - 300 | 10 | 8 | 7 | 11 | 0.69 | 0.85 | 0.50 | 0.73 | 0.59 | 0.86 |
| EMBRA216 | 2 | 200 - 250 | 7 | 6 | 3 | 10 | 0.40 | 0.79 | 0.67 | 0.77 | 0.55 | 0.84 |
| EMBRA219 | 1 | 245 - 280 | 9 | 10 | 7 | 12 | 0.63 | 0.84 | 0.63 | 0.84 | 0.63 | 0.87 |
| EMBRA222 | 1 | 75 - 93 | 11 | 7 | 6 | 12 | 0.80 | 0.88 | 0.81 | 0.77 | 0.81 | 0.85 |
| EMBRA226 | 7 | 160 - 210 | 13 | 10 | 9 | 14 | 0.69 | 0.90 | 0.50 | 0.86 | 0.59 | 0.90 |
| EMBRA227 | 3 | 280 - 320 | 9 | 8 | 7 | 10 | 0.69 | 0.78 | 0.75 | 0.82 | 0.72 | 0.81 |
| EMBRA233 | 6 | 155 - 185 | 7 | 8 | 7 | 8 | 0.25 | 0.68 | 0.50 | 0.73 | 0.38 | 0.80 |
| EMBRA240 | 8 | 230 - 260 | 7 | 9 | 7 | 9 | 0.44 | 0.78 | 0.87 | 0.85 | 0.65 | 0.85 |
| Mean ± S.E. | | | 10.7±3.1 | 10.7±2.6 | 7.3±2.0 | 14.1±3.8 | 0.63±0.19 | 0.84±0.07 | 0.68±0.15 | 0.85±0.05 | 0.66±0.14 | 0.88±0.04 |

^a^ Microsatellite EMBRA94 maps to linkage group 6 with LOD<3.0 and therefore does not appear on the linkage map.

.

.
